# Diagnosis and identification of *Leishmania* species in patients with cutaneous leishmaniasis in the state of Roraima, Brazil's Amazon Region

**DOI:** 10.1186/s13071-020-04539-8

**Published:** 2021-01-07

**Authors:** Joseneide Viana de Almeida, Cristian Ferreira de Souza, Andressa Alencastre Fuzari, Christie A. Joya, Hugo O. Valdivia, Daniella Castanheira Bartholomeu, Reginaldo Peçanha Brazil

**Affiliations:** 1 Programa de Pós-Graduação em Biologia Parasitária, Instituto Oswaldo Cruz/Fundação Oswaldo Cruz, Rio de Janeiro, RJ Brasil; 2 Laboratório de Doenças Parasitárias, Instituto Oswaldo Cruz/Fundação Oswaldo Cruz, Rio de Janeiro, RJ Brazil; 3 Department of Parasitology, US Naval Medical Research Unit 6, Lima, Peru; 4 Departamento de Parasitologia, ICB, Universidade Federal de Minas Gerais, Belo Horizonte, Minas Gerais Brazil

**Keywords:** Cutaneous leishmaniasis, *Leishmania* spp., Molecular diagnosis, PCR

## Abstract

**Background:**

Cutaneous leishmaniasis (CL) is an endemic disease in Brazil that is highly prevalent in the northern region of the country. Although there is a continuous and growing number of cases registered in the state of Roraima, there is limited information regarding the species of *Leishmania* that affect the human population. In this study, we aimed to characterize which *Leishmania* species cause human disease in those presenting with cutaneous leishmaniasis in endemic areas of the State of Roraima.

**Methods:**

We conducted a prospective surveillance study between 2016 to 2018 in health centers located in the State of Roraima, Brazil. Participants with clinical suspicion of CL were enrolled and provided lesion samples for parasitological detection by microscopy. A subset of the samples was tested by polymerase chain reaction and sequencing of the internal transcribed spacer 1 (ITS-1 PCR) for molecular species identification.

**Results:**

A total of 262 participants were enrolled in this study. Of those, 129 (49.27%) were positive by parasitological examination. Most positive subjects (81.58%) were male, and most cases presented a single lesion (80.26%). ITS-1 PCR and sequencing on a subset of 76 samples allowed us to detect nine different species of *Leishmania*: *L. (V.) braziliensis*, *L (V.) panamensis*, *L. (V.) guyanensis*, *L. (V.) naiffi*, *L. (V.) shawi*, *L.(V.) utingensis*, *L. (V.) lindenbergi, L. (L.) amazonensis* and *L. (L.) mexicana*.

**Conclusions:**

Our study provides the first assessment of circulating species of *Leishmania* in the State of Roraima, Brazil, and shows the high diversity in this region. This study opens the path for further research on the transmission of leishmaniasis in the northernmost Brazilian State including vector and reservoir surveillance as well as for intensification of investigation and control activities against CL in the region.
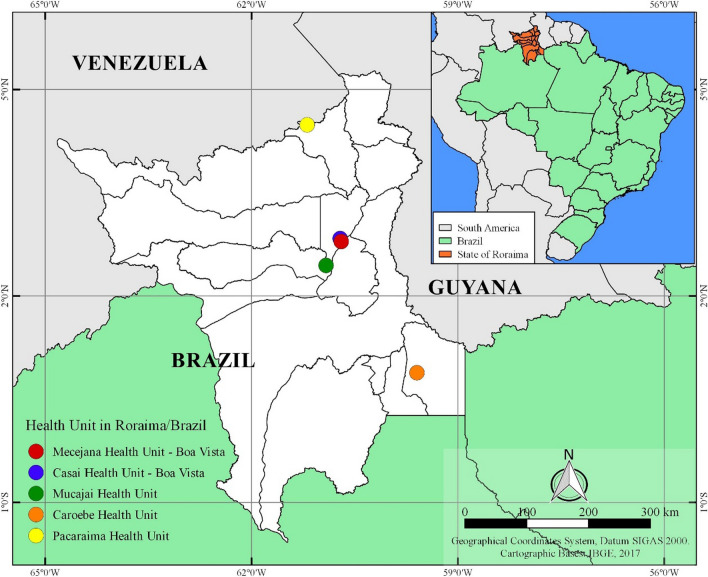

## Introduction

Leishmaniasis is a complex of diseases caused by species of intracellular protozoa, morphologically similar, of the genus *Leishmania* (Kinetoplastida: Trypanosomatidae) and transmited by the bite of infected female sand flies (Diptera: Psychodidae) [1]. The disease is highly prevalent in poor countries and vulnerable populations with limited access to health services [2]. In 2017, 94% of all new cases reported to the WHO occurred in seven countries: Brazil, Ethiopia, India, Kenya, Somalia, South Sudan and Sudan, and approximately 90% of mucocutaneous leishmaniasis cases occurred in Bolivia, Brazil and Peru [3].

Leishmaniasis is highly prevalent in the northern region of Brazil where it accounts for a large proportion of cases each year [3]. The Brazilian Amazon is in this area [3, 4] and is comprised of municipalities of large territorial extension and difficult access. This scenario poses challenges to implement measures recommended by the Brazilian Ministry of Health for the control of CL and is further complicated by the multiple ethnic, cultural and environmental factors that characterize this region [4, 5]. Previous studies have shown that Brazil’s Amazon region has undergone profound environmental changes resulting from mining, agriculture and livestock expansion [6], which may be contributing to changes and maintenance of the leishmaniasis cycle in the area. The State of Roraima, which is in this region, has experienced these changes with an increase of leishmaniasis cases between 2011 to 2015 [3]. Recorded data on CL cases through the Ministry of Health's Information System for Notifiable Diseases (SINAN) [7] between the years 2008 to 2018 revealed that the average number of cases reported in Roraima was 3477 cases per year.

Studies describing the epidemiological profile of the disease in Roraima have shown that the majority of reported cases are in men, of working age and migrants involved in activities such as deforestation and occupation of areas such as settlement projects [8]. The predominant clinical form of the disease is cutaneous with one single lesion; regarding treatment, most of these patients were discharged for healing [3].

To understand CL transmission in the State of Roraima, the present study focused on identifying CL in patients from the public health network of Roraima as well as performing molecular diagnosis by PCR (polymerase chain reaction) and sequencing for *Leishmania* species determination. The results obtained provide information that will help to develop measures for interventions and efficient control strategies for human CL in the state.

## Materials and methods

### Study area

**Fig. 1:**
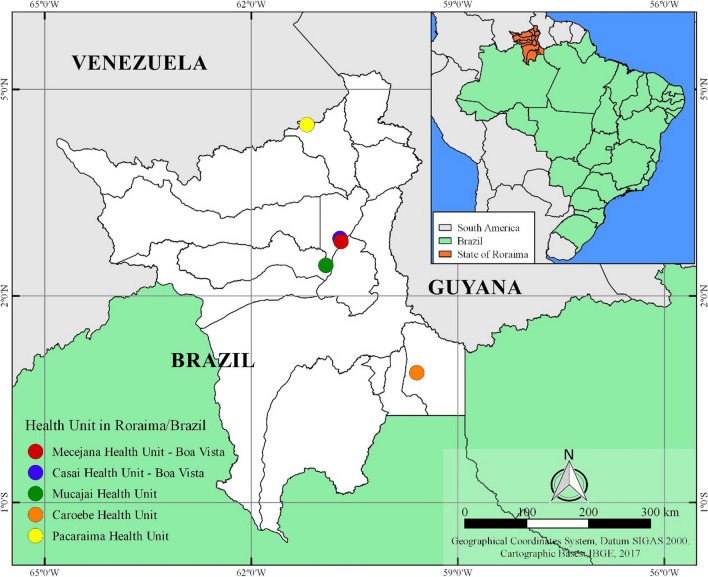
Distribution of collection sites according to municipalities of Roraima. The inset shows in the left the number of samples collected as part of this study at each laboratory. The map on the right side shows the location of the State and the study sites

The State of Roraima was created in 1988 and belongs to the North region of Brazil. The state shares borders to the south with the State of Amazonas, to the East with the Republic of Guyana and the State of Pará and to the West with the State of Amazonas and Venezuela. It has a territorial area of 224,299 km^2^ that is divided into 15 municipalities: Alto Alegre, Amajarí, Boa Vista (Capital), Bonfim, Cantá, Caracaraí, Caroebe, Iracema, Mucajaí, Normandy, Pacaraima, Rorainópolis, São João da Baliza, São Luiz and Uiramutã (Fig. 1) [9]. The estimated population is 605,761 inhabitants including Venezuelan immigrants [10].

### Patient selection and sample collection

The study included patients with suspected CL that were enrolled between 2016 to 2018 at the Reference Laboratory of the Mecejana Health Center and at the Indian Health House (CASAI) in Boa Vista and different Health Units in the municipalities of Mucajaí, Caroebe (southern region of Roraima state) and Pacaraima on the border with Venezuela. Patients were informed about the research and provided informed consent. A clinical examination was performed in all patients and a questionary was administered to collect socioeconomic and demographic data (sex, age group), presence of lesions, previous leishmaniasis episodes and treatment history.

Samples were collected from these patients by scraping of the lesion for microscopy. Remaining tissue was used to prepare filter paper imprints for molecular detection of *Leishmania* species. The study was approved by the ethics committee of the Federal University of Roraima under protocol number CAAE 57445116.3.0000.5302.

### Parasitological analysis

Direct examination was performed by collecting tissue at the edge of the ulcerated lesion using an aseptic technique with a lancet and/or sterile scalpel. The material was spread onto microscopy slides, fixed with methanol and stained with Giemsa and/or Panotic. The slides were read using optical microscopy at 100× magnification [1].

### Molecular studies

After collecting the samples on filter paper, DNA extraction was performed using the Gentra Puregene Tissue Kit (QIAGEN®), following the manufacturer's protocol. PCR of the internal transcribed spacer 1 was performed using primers LITSR (5'CTGGATCATTTTCCGATG3') and L5.8S (5'TGATACCACTTATCGCACTT3') as previously described [11].

The PCR reaction was prepared under the following buffer conditions: 1× buffer solution (200 mM Tris-HCl pH8.4, 500 mM KCl), 1.5 mM MgCl_2_, 0.2 mM mixture dNTPs, 0.5 pmol of the LITSR primer, 0.5 pmol of the L5.8S primer, 1 U of Taq Platinum polymerase DNA (Invitrogen®) and 5 μl of template DNA, in a final volume of 25 μl. The amplification reaction was carried out by 33 cycles of denaturation at 95 °C for 30 s, annealing at 53 °C for 1 min and extension at 72 °C for 1 min in an automatic DNA thermocycler (MaxyGene Gradient, AXYGENE®). The reaction generates a fragment of approximately 350 bp, which was analyzed on a 2% agarose gel stained with GelRed™ and compared with controls of *L. (V.) braziliensis* (MHOM/BR/75/M2903).

PCR-positive samples were sent to Macrogen® (Seoul, Korea) for bidirectional Sanger gene sequencing using an automatic sequencer (Applied Biosystems 3730XL). The obtained sequences were aligned and analyzed using the Sequencher®4.1.4 program and subsequently compared by BLAST and deposited in GenBank (sample ID: MT606220-MT606276) [12].

### Data analysis

To provide additional confirmation to the species detected by Blast, the resulting Fast sequences were used for discriminant analysis of principal components. Briefly, reference sequences from *L. (V) panamensis* PSC1, *L. (V) braziliensis* (LH2215), *L. (L) amazonensis* (M2269) and *L. (L) mexicana* (LEM2284) strains were downloaded and aligned with the clinical sequences from our study using Clustal omega [13].

The resulting multiple sequence alignment was loaded into R using the package “ape” [14] and cleaned prior to genetic analysis using the package “poppr” in order to secure that only high quality data remains [15]. The parameter used excluded positions with > 30% missing calls. DAPC was performed on the cleaned dataset using the R “adegenet” package [16]. Data obtained in the study were organized into a Microsoft Excell 2013® spreadsheet and used for data analysis in GraphPad Prism 5.0 software (GraphPad Software Inc., San Diego, CA, USA). Due to the categorical nature of the samples, data were analyzed using the chi-square test (x²) on BioEstat 5.0 [17] with a significance level of 95% (α = 0.05).

## Results

**Fig. 2:**
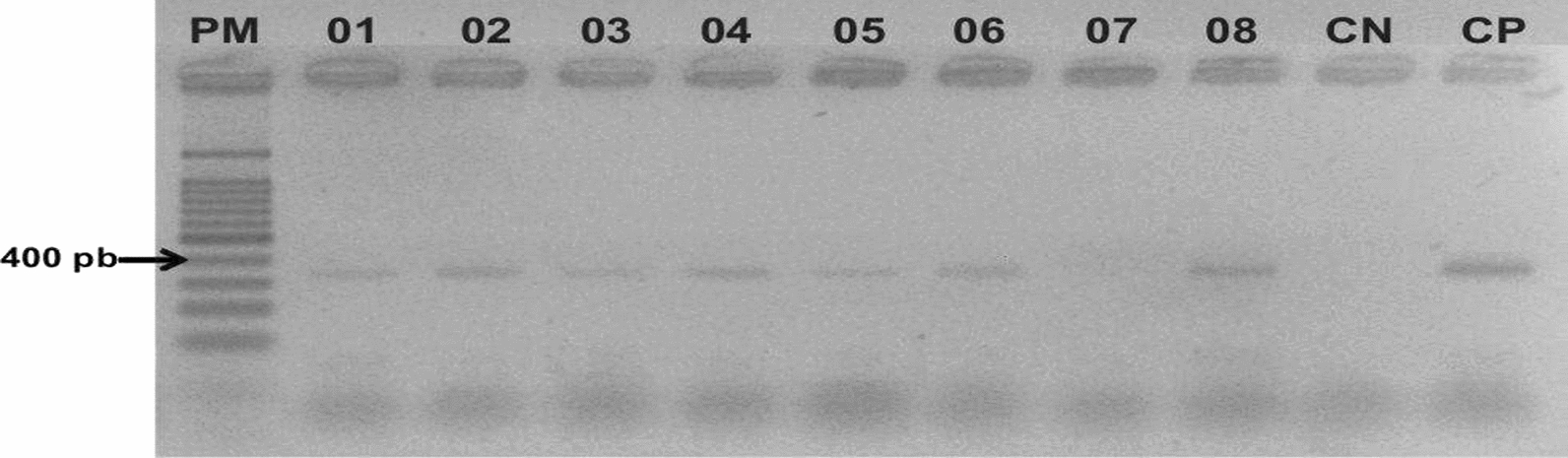
PCR results of *Leishmania* spp. on filter papers of suspected CL patients in Roraima in 2% agarose gel stained with Red gel. PM: base pair marker (100bp); CN: negative control (pure DNA-free water); CP: positive control of *Leishmania braziliensis* (MHOM/BR/75/M2903); 07: negative sample; 01-06 and 08: positive samples

In the period between 2016 and 2018, 262 samples were collected from patients with suspected CL in the municipalities of Boa Vista, Mucajaí, Caroebe, Pacaraima and CASAI in Roraima (Fig. 1). Parasitological and PCR tests showed that 129 samples (49.27%) were positive for *Leishmania* sp. (Fig. 2). Of these samples, a subset of 76 samples were sent for sequencing.

Sequencing results allowed the identification of *Leishmania* species distributed in nine municipalities in the state of Roraima.

Of 76 patients, 67 (88.16%) were from rural areas and 9 (11.84%) from urban settings (Tables 1 and 2). In the analysis regarding the gender distribution, 81.58% (*n* = 62) of all cases were in men (Table 1). All patients presented the clinical cutaneous form with the following distribution of lesions: 80% (*n* = 61) with a single lesion, 9% (*n* = 7) with two lesions and 11% (*n* = 8) with three or more lesions. In the assessment by age group the highest percentage of cases occurred in patients between 21 to 40 years (60.5%, *n* = 46).

**Table 1:** Distribution of *Leishmania* species identified in the sequencing of the material obtained by PCR-ITS-1 by sex, age, area of origin and nationality in the State of Roraima, Brazil, from 2016 to 2018

Patient/variables	Number of samples	1 lesion	2 lesions	≥ 3 lesions	DNA (+)	*Leishmania* species identified by sequencing									
						L.b	L.a	L. g	L.p	L.s	L.l	L.u	L. sp.	L. n	L.m
Gender															
Male	62														
Female	14														
Age range															
0 to 10	4	4			4	2			1				1		
11 to 20	6	5		1	6		2		1	2			1		
21 to 30	18	13	2	3	18	5	4		4	1	1		3	1	
31 to 40	28	20	5	2	28	11	4	3	3				6		
41 to 50	13	13		1	13	3	3		1			1	5		
≥ 51	7	6		1	7	2	2	1					2		
Area															
Urban	9	6	2	1	9	3	2		1				3		
Rural	67	56	5	6	67	20	12	4	9	3	1	1	15	1	1
Origin By nationality															
Brazil	71	60	7	7	71	23	14	4	11	3	1	1	13	1	
Venezuela	3	3	0	0	3	0	1						2		
Guyana	2	2	0	0	2	0		1					1		
Indigenous	6	3	1	2	6	3			2	1					

*L.b* = *Leishmania braziliensis*; *L. a* = *Leishmania amazonensis*; *L.g* = *Leishmania guyanensis*; *L.p* = *Leishmania panamensis*; *L. s* = *Leishmania shawi*; *L.l* = *Leishmania lindenbergi*; *L. u* = *Leishmania utingensis*; *L. sp.* = *Leishmania sp.*; *L.n* = *Leishmania naiffi; L.m= Leishmania mexicana.*

**Table 2:** Distribution of *Leishmania* species identified by OTS1 sequencing according to the municipality of origin of patients

Municipalities/Country	L. b	L. a	L.g	L. n	L.s	L.p	L.u	L. sp.	L. l	L.m
Species										
Boa Vista	3	2						4		
Alto Alegre	7		2			3			1	1
Cantá		5		1						
Caroebe	1	1	1		2			4		
Mucajaí	3	1					1			
Iracema	2	1								
São Luiz		1								
Pacaraima		1				2		4		
Amajarí	8				1	5		2		
Pres. Figueredo - AM								1		
Guyana			1					1		
Venezuela		1						2		
TOTAL	24	13	4	1	3	10	1	18	1	1

*L.b* = *L. braziliensis*; *L. a* = *L. amazonensis*; *L.g* = *L. guyanensis*; *L.p= L. panamensis*; *L.s* = *L. shawi*; *L.l* = *L. lindenbergi*; *L. u* = *L. utingensis*; *L. sp.* = *Leishmania sp.*; *L.n* = *L. naiffi*; *L.m* = *Leishmania mexicana*

This study detected six of the seven species of *Leishmania* found by the Ministry of Health to cause CL in Brazil. In addition, we also observed the occurrence of *L. (L.) mexicana* and *L. (V) panamensis* (Table 2)*.*

**Fig. 3:**
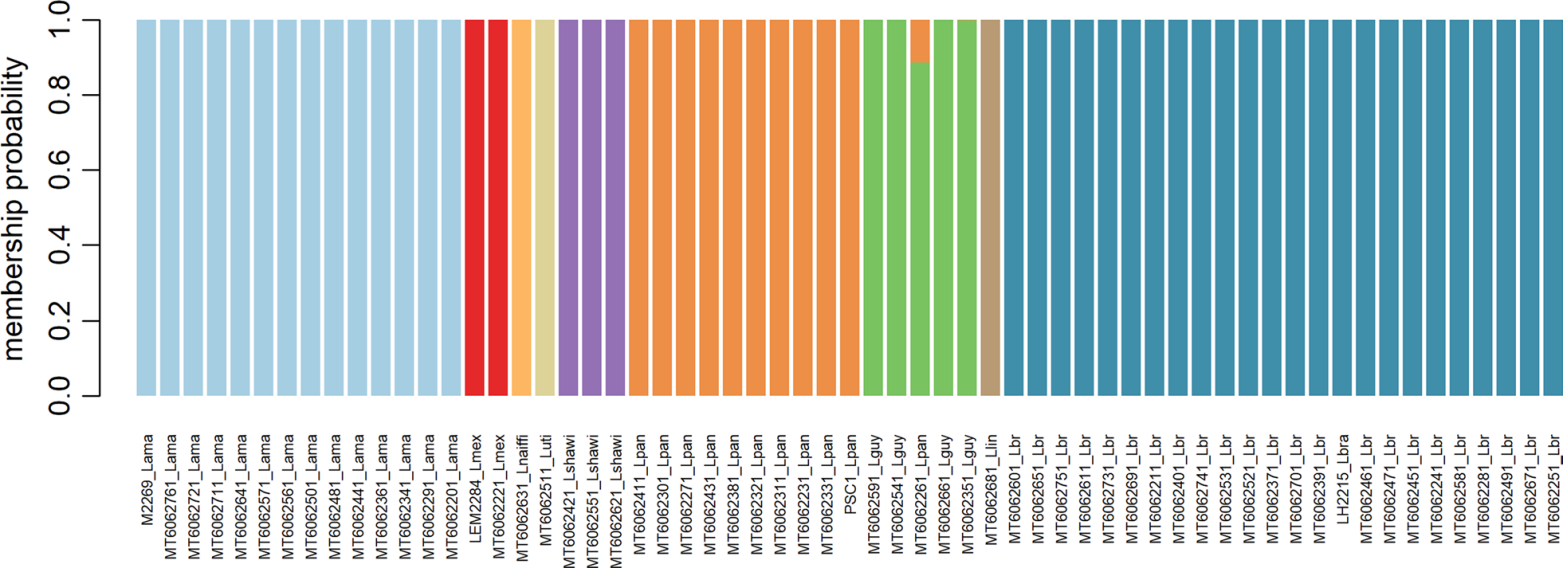
Population structure. The figure shows the population structure of the *Leishmania* samples collected from Roraima. The majority of samples clustered closely to their reference sequences (*L. panamensis* PSC1, *L. braziliensis* LH2215, *L. amazonensis* M2269 and *L. mexicana* LEM2284 strains) or in separate groups according to the species suggested by Blast

The PCA provided further support to the GenBank results and was fully able to cluster most clinical samples with their species reference sequences (Fig. 3). In this regard, nine out of ten putative *L. (V.) panamensis* samples clustered with the *L. (V.) panamensis* PSC1 reference whereas the remaining sample (MT6062261) clustered with the *L. (V.) guyanensis* samples. In addition, the *L. (L.) mexicana*, *L*. *(V.) braziliensis* and *L. (L.) amazonensis* clinical samples clustered with their respective reference sequences *L. (L.) mexicana* (LEM2284), *L. (V.) braziliensis* (LH2215) and *L. (L.) amazonensis* (M2269). Furthermore, *L. (V.) naiffi*, *L. (V.) shawi*, *L. (V) lindernbergi* and *L. (V) utingensis* presented separate clusters supporting their GenBank identification (Fig. 3)

*L. (V) braziliensis* was the most frequent species in our samples accounting for 31.6% of the 76 samples (*n* = 24), followed by *L. (L) amazonensis* with 17.0% (*n* = 13), *L. (V) panamensis* with 13.2% (*n* = 10), *L. (V) guyanensis* with 5.3% (*n* = 4), *L. (V) shawi* with 3.9% (*n* = 3) and *L. (V) naiffi, L. (V) utingensis, L. (V) lindenbergi* and *L. (L) mexicana* with 1.31% (*n* = 1) each. The remaining samples (*n* = 18, 23.7%) presented profiles without specific identification.

Regarding the *Leishmania* species identified in the municipalities of Roraima, we observed that 31.38% (*n* = 24) were *L. (V.) braziliensis* present in samples from patients in the municipalities of Boa Vista, Alto Alegre, Caroebe, Mucajaí, Iracema and Amajarí, (Table 2). *L. (L.) amazonensis* was identified as the second most characterized species in this study in 18.4% (*n* = 14) of the samples, distributed in the municipalities of Cantá, Boa Vista, Alto Alegre, Caroebe, Mucajaí, São Luiz and Amajarí and in Venezuela with one case. In 13.15% (*n* = 10) of the samples, *L. (V.) panamensis* was distributed in the municipalities of Amajarí, Pacaraima and Alto Alegre. In 6.57% (*n* = 5) of the samples *L. (V.) guyanensis* was detected in the municipalities of Alto Alegre and Caroebe and in Guyana. *L. (V.) shawi* was identified in 3.94% (*n* = 3) of the samples from the municipalities of Caroebe and Amajarí. L. (V.) naiffi was identified in a patient sample from the municipality of Cantá, *L. (V.) utingensis* was detected in an individual residing in the municipality of Mucajaí, and *L. (V.) lindenbergi* and *L. (L.) mexicana* were detected in Alto Alegre with one case for each species**.**

## Discussion

The study showed that, although most positive cases were from residents in Brazil, we had cases of patients residing in Guyana and Venezuela. In this regard, reports from the health professionals who attended the patients showed that many individuals come from neighboring countries and cross the border with Brazil in search of diagnosis and treatment.

The study also revealed six positive cases in indigenous subjects of different ethnic groups, corroborating the work of Pezente who described the epidemiological profile of CL in the State of Roraima between the years 2007 and 2016 [8]. That study showed an incidence of CL of 13% in indigenous populations, demonstrating the need for further studies to understand the impact of CL in this population.

In the present study, most patients with clinical suspicion of CL were in people living in rural areas. The rate of positivity for *Leishmania* was higher in males and in the age group between 21 and 40 years old, which represented 60.3% of cases (*n* = 46). This is an important finding, since this demographic group represents most of the economically active population.

This case profile is like those observed in a previous epidemiological study conducted in the state [15], which observed that most cases occured in people between 31.6 to 34.5 years old [18, 19]. The results also demonstrate a predominance of single CL lesions, which is in accordance with other studies conducted in the Amazon [19, 20] and Pará [21].

Regarding the distribution by municipalities, our study showed that all cases were distributed in 9 of the 15 municipalities in the state of Roraima. Although cases covered all the State microregions [9], > 55% of them were concentrated in the municipalities of the Boa Vista microregion. This could be related to the fact that this region has active mining areas and is among the first entry points for immigrants coming from Venezuela.

Our GenBank and PCA results provide support for the introduction of *L. (V) panamensis* in Brazil as well as the potential introduction of *L. (L.) mexicana.* The presence of one *L. (V.) panamensis* sample that clustered closer to *L. (V.) guyanensis* requires additional confirmation by other methods such as multilocus sequence typing or next generation sequencing. Furthermore, additional analyses are needed to confirm the finding of *L. (V.) naiffi, L. (V.) shawi*, *L.(V) lindernbergi* and *L. (V) utingensis* because of the lack of whole genomes or reference sequences from these species.

A subset of sequenced samples (*n* = 18) was not identified to the species level and remained as *Leishmania* sp. These sequences presented a low quality, which limited subsequent alignment, blast and PCA analysis.

Our study showed that most cases were due to *L. (V.) braziliensis*, which was identified in 31.6% (*n* = 24) of the samples. In Brazil, this species is the main causative agent of CL with a recent report of expansion in the Amazon [22, 23]. Our findings suggests that this species may be circulating and causing the disease in all municipalities in the State, as it was identified in at least one municipality of each micro-region. This assumption is supported by a previous study on phlebotomine fauna in the municipality of Caroebe [24] in the southern part of the state where *Lutzomyia davisi* was the most abundant species in the primary forest. This sand fly species has been found naturally infected with *L. (V.) braziliensis* in several locations in the Brazilian Amazon [25].

*Leishmania (L) amazonensis* was the second most identified species and was detected in the municipalities of Boa Vista, Alto Alegre, Cantá, Caroebe, Mucajaí, São Luiz and Amajarí and in Venezuela. As observed in other states of the Amazonian region [19, 22], this species has an important role in public health, since it is associated with diffuse cutaneous leishmaniasis (DCL), which induces anergy in the individual's immune cell response. In addition, there have been reports of mucosal cases caused by this parasite species [1, 26]. The presence of *Lutzomyia flaviscutelata* in Serra do Tepequém of the Municipality of Amajarí [5] reinforces the transmission of *L*. *(L) amazonensis* in this region as observed in the Brazilian Amazon [27].

In the case of *L. (V.) guyanensis*, there are previous reports of the presence of its vector, *Lutzomyia (Nyssomyia) umbratilis,* in Serra do Tepequém in Amajarí [5], and it is known that this species has a wide distribution in areas of primary forest in the Amazon Region. Although infrequent, the occurrence of *Lutzomyia (Nyssomyia) umbratilis* in Serra do Tepequém should be a warning sign, since contact with this species in forest areas generally results in the transmission of *Leishmania (V.) guyanensis* to humans.

The presence of *L. (V.) guyanensis* in samples in the cities of Alto Alegre, Caroebe and in Guyana supports this finding and demonstrates that this species may be circulating on a larger scale in the state. Clinically, individuals affected by this species may present single or multiple lesions, the latter being more frequently due to several vector bites or lymphatic metastases, with the possibility of lymphangitis [28]. This species was also identified in mucosal lessions in the state of Rondônia [29] expanding the possibility of mucosal clinical presentation.

Our study reports for the first time the presence of *L. (V.) panamensis* in Roraima with the detection in the municipalities of Alto Alegre, Pacaraima and Amajarí, located in the northern region of the state. *L. (V) panamensis* is the main causative agent of cutaneous leishmaniasis in Panama [30] and Colombia and is responsible for a relatively large number of cases in other neighboring countries with records of approximately 3000 new cases per year [31].

We also detected the presence of *L. (V.) shawi* in two human cases. This species was detected in monkeys from the species *Cebus appela* and *Chiropotes satanus*, sloths *Choloepus didactylus* and *Bradypus tridactylus,* procyonid *Nasua nasua* and in the sand fly *Lutzomyia whitmani*, all of them from primary forest areas of the State of Pará [32].

The presence of *L. (V.) naiffi* in human CL cases in Roraima complements previous reports from the States of Amazonas [33, 35] and Pará [34], which indicates that this species is not uncommon in the region.

Two other species of *Leishmania* that were detected in this study were *L. (V.) utingensis* and *L. (V.) lindenbergi*, which accounted for one case each. *L (V) utingensis* was first reported in 1977 from an infected *Lutzomyia tuberculata* from the Brazilian State of Pará [36]. The scarcity of data on this species as causative CL agent in humans underscores the need of further research to characterize this species.

*L. (V) lindenbergi* was reported in soldiers deployed in forested areas in the City of Belém of the Brazilian State of Pará [37] and more recently in two CL cases in the state of Rondonia [38]. In the study, we also observed the occurrence of *Leishmania (L.) mexicana*, which is a species commonly found in Central America and in the northern region of South America [26].

## Conclusions

We provide evidence that several *Leishmania* species are present in the State of Roraima infecting local people as well as foreign and Brazilian migrants that are circulating not only in the state but also in the Amazon region. These results are important as they open an opportunity for additional research related to disease incidence, treatment responses, circulating reservoirs and vectors, which are key components for the development of effective control programs.

## Data Availability

All data generated or analyzed during this study are included in this article and its additional file. Raw data are available upon request to the corresponding author.

## Abbreviations

LC: Cutaneous leishmaniasis
SINAN: Information System for Notification of Complaints
PCR: Polymerase chain reaction
WHO: World Health Organization
MS: Ministry of Health
PDL: Direct parasitological examination
CASAI: Indian health home
LCD: Diffuse cutaneous leishmaniasis
